# A Case of Metaplastic Breast Cancer with Prolonged Response to Single Agent Liposomal Doxorubicin

**DOI:** 10.7759/cureus.454

**Published:** 2016-01-11

**Authors:** Lamya Hamad, Thaer Khoury, Karen Vona, Jill Nestico, Mateusz Opyrchal, Kilian E Salerno

**Affiliations:** 1 Pharmacy, Roswell Park Cancer Institute; 2 Pathology, Roswell Park Cancer Institute; 3 Soft Tissue Medicine, Roswell Park Cancer Institute; 4 Medical Oncology, Roswell Park Cancer Institute; 5 Radiation Medicine, Roswell Park Cancer Institute

**Keywords:** metaplastic carcinoma, oncology, brca1, anthracyclines, breast cancer, triple negative breast cancer

## Abstract

Female breast cancer accounts for 14% of all new cancer cases in the United States. Metaplastic breast carcinomas (MpBC) are less than 1% of all mammary tumors. Limited clinical information exists on the prognosis and treatments for MpBC due to its rarity and the histological diversity of the tumor cells.

We report a case of metastatic MpBC with osseous differentiation who had a marked response to liposomal doxorubicin. This 54-year-old female presented with a 2.5 x 2.6 cm oval-shaped irregularly marginated density in the outer lower quadrant of the left breast, cT2N0M0. Biopsy revealed an invasive metaplastic carcinoma, triple negative, with osseous and focal chondroid differentiation. Genetic testing showed a mutation in the BRCA1 gene. Approximately seven months after a left mastectomy, the patient presented with biopsy-proven metastatic disease and was initiated on therapy with a single agent, liposomal doxorubicin, 50 mg/m^2^ every 28 days. The patient achieved maximum response after cycle three and continued to have stable disease for 18 months (20 cycles). Based on the pathologic phenotype, we would have predicted that she would have had a poor and short response to anthracycline. This case illustrates an increased sensitivity of BRCA1-mutated cancers to anthracycline therapies, irrespective of pathologic classification.

## Introduction

Female breast cancer accounts for 14% of all new cancer cases in the United States [1]. Metaplastic breast carcinomas (MpBC) are seen in less than 1% of all mammary tumors [2-3]. Limited clinical information on the prognosis and treatments exists for MpBC due to its rarity and the histological diversity of the tumor cells [1-4]. Metaplastic carcinomas are characterized by a combination of various cell types, most commonly spindle cell, squamous, and/or mesenchymal [2, 5]. Some studies suggest that these cells may have developed from multipotential undifferentiated cells [2].

Treating patients with MpBC presents unique challenges. Song, et al. found that patients with MpBC presented with a larger tumor size, fewer lymph node metastases, a higher percentage of triple-negative (estrogen receptor-negative, progesterone receptor-negative, and human epidermal growth factor receptor-2-negative) cases, higher proliferation rates, and high expression of Ki-67 compared to those with invasive ductal carcinomas (IDC) [6]. Patients with MpBC have a poorer prognosis and are more likely to have local recurrences than IDC patients, even when compared only to patients with triple-negative IDC [6].The study also found that patients with MpBC were likely to develop distant metastases, specifically to the lungs [6]. The five-year survival rate in the MpBC group versus IDC was found to be 54.5% and 85.1%, respectively, whereas the five-year disease-free survival rate was 45.5% in MpBC patients versus 71.2% in the IDC group with 60.3% in triple-negative invasive carcinomas [6].

Typically, patients with MpBC are treated similarly to those with IDC; however, studies have shown reduced response rates to chemotherapy among MpBC patients compared to IDC patients [3, 7]. Rayson, et al. found that standard chemotherapy regimens in the adjuvant setting are not as effective with MpBC compared to other types of breast cancers [3]. Of seven patients treated using a total of 14 separate chemotherapy regimens, only one response was observed to doxorubicin, with this patient exhibiting stable disease for 12 months [3]. The patient population in the Rayson, et al. study was predominantly metaplastic breast carcinomas with pure spindle cell differentiation, which may limit the generalizability of the study to other subtypes of MpBC.  

## Case presentation

**Figure 1:**
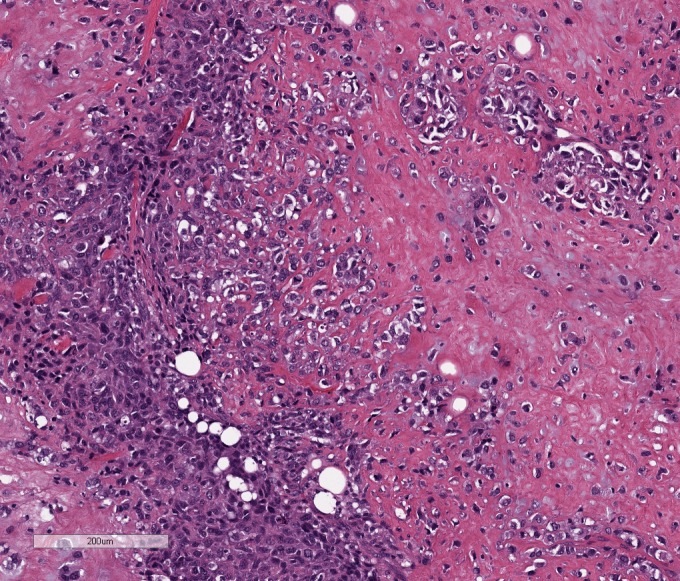
Metaplastic mammary carcinoma with osseous differentiation (Hematoxylin and Eosin 10x)

A 54-year-old female presented with a 2.5 x 2.6 cm oval-shaped irregularly marginated density in the outer lower quadrant of the left breast with an irregular calcification within the mass. Informed patient consent was obtained. A biopsy revealed an invasive metaplastic breast carcinoma, triple-negative, with osseous and focal chondroid differentiation. A left mastectomy with sentinel node biopsy was performed. Pathology revealed a tumor size of 5.8 x 5.6 x 4.7 cm, margins were free of malignancy, and two sentinel lymph nodes were negative for tumor, pT3N0(sn)M0. The patient declined adjuvant therapy and proceeded with surveillance. Approximately four months later, a CT scan of the chest, abdomen, and pelvis revealed multiple new parenchymal lung nodules. The patient opted for continuing surveillance, and follow-up imaging three months later revealed a progressive increase in the size of the pulmonary nodules, highly concerning for metastases. The patient then presented to our oncology clinic. Lung biopsy confirmed high-grade malignancy similar to her initial diagnosis consistent with metastatic MpBC (Figure 1). Estrogen receptor, progesterone receptor, and HER2 protein were once again not expressed.

**Figure 2:**
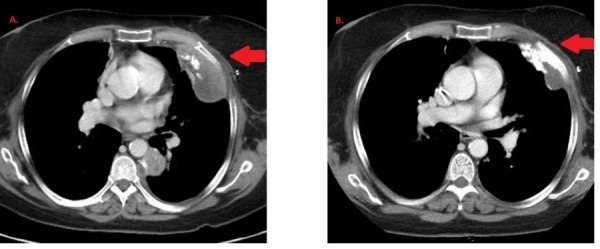
Representative picture of lung lesion at first restaging scan

Treatment decision making took into consideration the rarity of MpBC, the lack of well-defined standard therapy, and that systemic therapy usually mimics that of IDC [7]. Doxorubicin is a preferred treatment option for both triple-negative breast cancers as well as many soft tissue sarcomas. Thus, the patient was initiated on systemic therapy with a single agent, liposomal doxorubicin, 50 mg/m^2^ every 28 days. A restaging CT of the chest, abdomen, and pelvis with contrast was done approximately every three months. Partial response was achieved on the first re-staging scan (Figure 2). As she developed hand and foot syndrome, dosing was extended to every five weeks with better tolerance.

**Figure 3:**
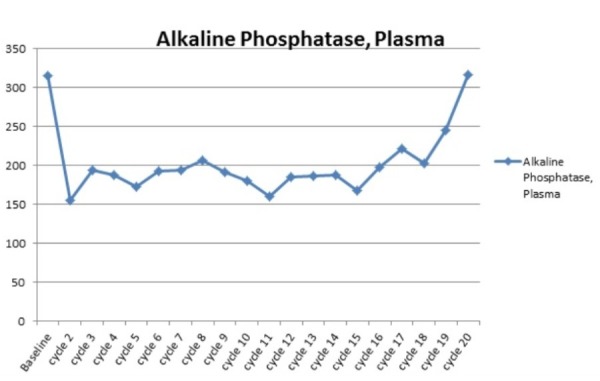
Levels of alkaline phosphatase (IU/L) at baseline and during the course of therapy. Levels corresponded to her radiological response, with initial partial response followed by stable disease

The patient achieved maximum response after cycle three and continued to have stable disease for 18 months (20 cycles) before observing a mild progression of multiple pulmonary nodules and pleural masses. The patient’s alkaline phosphatase levels corresponded to the disease response, leading us to believe it was most likely produced by the cancer cells, as there was no evidence of bone invasion (Figure 3). Given the progression, therapy was changed to capecitabine, due to patient preference for oral therapy, without response. She is currently being evaluated for treatment on a clinical trial including PARP inhibitors or therapy with a platin-containing regimen. Five months after initiating therapy with liposomal doxorubicin, genetic testing by FoundationOne was performed to provide additional information for determination of options for future therapies. The FoundationOne report listed a genomic alteration in the BRCA1 gene, G511fs*21 mutation, which is expected to result in the truncation of the protein resulting in the loss of two BRCT (BRCA1 C-terminus) domains and, therefore, it was predicted to result in abrogation of function. The patient preferred to not have germline BRCA1 testing done; therefore, it is unknown if it is a germline mutation, although she does not have any family history of malignancies.

## Discussion

This case study presents a patient with triple-negative metastatic MpBC who had a prolonged response to liposomal doxorubicin. The initial treatment decision was based on the efficacy of anthracycline-based regimens in sarcomas and metastatic breast cancer. Some studies have explored the association between BRCA1 and BRCA2 mutations in tumors and sensitivity to anthracycline-based regimens [8-9]. The BRCA1 protein helps repair strand breaks in the DNA [10]. There are more than 1,800 mutations in the BRCA1 gene, many of which have been associated with an increased risk of breast cancer [10]. Doxorubicin interferes with DNA and RNA synthesis and inhibits topoisomerase-II at the point of DNA cleavage, resulting in DNA strand breaks. BRCA-deficient cancer cells are less able to repair damage caused by DNA-damaging cytotoxic chemotherapy agents; therefore, they are more sensitive to these agents [8]. A retrospective multicenter study involving 155 patients treated with second or third-line liposomal doxorubicin monotherapy or combined with platinum found that BRCA1 mutation carriers had longer median time to treatment failure, longer overall survival, and a better response rate [8]. The study concluded that among epithelial ovarian cancers, patients with defective BRCA1 genes are more sensitive to DNA-damaging agents as compared to other epithelial ovarian cancers [8]. In another study, Graeser, et al. found a correlation between functional homologous recombination deficiency and chemosensitivity to anthracycline-based regimens in sporadic breast cancer [9].  

This patient was found to have a G511fs*21 mutation of the BRCA1 gene, a missense mutation which most likely resulted in a decrease of functional protein. The G511fs*21 mutation has not been previously described, and since the patient did not have germline testing, it is unknown if it is germline or cancer-specific mutation. Germline BRCA1 mutations impair the DNA repair pathway, resulting in the accumulation of genetic defects that ultimately lead to the development of tumor cells [10].  

In general, MpBC has been associated with poor outcomes and chemoresistance. This case is unusual in its durable response to treatment with a single agent, liposomal doxorubicin. The BRCA1 mutation may have resulted in heightened chemosensitivity to anthracycline therapy. Based on the pathologic phenotype of this patient’s MpBC, we would have predicted a poor and short response to anthracycline. Therefore, genetic testing may be useful in choosing best treatment options for rare tumors without established standard therapies.

## Conclusions

This case illustrates increased sensitivity of BRCA1 mutated cancers to anthracycline therapies, irrespective of pathologic classification. Therefore, a mutational analysis might help to predict responses and choice of treatment in select patients. For this patient, other therapies based on BRCA1 deficiency could be chosen. Poly ADP-ribose polymerase (PARP) inhibitors and platinum-based chemotherapy should be strongly considered. She is currently being evaluated for treatment on a clinical trial including PARP inhibitors or therapy with a platin-containing regimen. More comprehensive studies exploring mutations in this rare tumor type might reveal novel treatment options for selected patients. This case highlights that genetic variations might be more predictive of response to therapy than histologic classification alone.
